# Syringin Prevents 6-Hydroxydopamine Neurotoxicity by Mediating the MiR-34a/SIRT1/Beclin-1 Pathway and Activating Autophagy in SH-SY5Y Cells and the *Caenorhabditis elegans* Model

**DOI:** 10.3390/cells12182310

**Published:** 2023-09-19

**Authors:** Ru-Huei Fu, Syuan-Yu Hong, Hui-Jye Chen

**Affiliations:** 1 Graduate Institute of Biomedical Sciences, China Medical University, Taichung 40402, Taiwan; dazingdog@hotmail.com; 2 Translational Medicine Research Center, China Medical University Hospital, Taichung 40447, Taiwan; 3 Ph.D. Program for Aging, China Medical University, Taichung 40402, Taiwan; 4 Department of Medicine, School of Medicine, China Medical University, Taichung 40447, Taiwan; 5 Division of Pediatric Neurology, China Medical University Children’s Hospital, Taichung 40447, Taiwan

**Keywords:** Parkinson’s disease, syringin, SH-SY5Y cells, 6-hydroxydopamine (6-OHDA), autophagy, Beclin-1, SIRT1, miR-34a, *C. elegans*, α-synuclein

## Abstract

Defective autophagy is one of the cellular hallmarks of Parkinson’s disease (PD). Therefore, a therapeutic strategy could be a modest enhancement of autophagic activity in dopamine (DA) neurons to deal with the clearance of damaged mitochondria and abnormal protein aggregates. Syringin (SRG) is a phenolic glycoside derived from the root of *Acanthopanax senticosus*. It has antioxidant, anti-apoptotic, and anti-inflammatory properties. However, whether it has a preventive effect on PD remains unclear. The present study found that SRG reversed the increase in intracellular ROS-caused apoptosis in SH-SY5Y cells induced by neurotoxin 6-OHDA exposure. Likewise, in *C. elegans,* degeneration of DA neurons, DA-related food-sensitive behaviors, longevity, and accumulation of α-synuclein were also improved. Studies of neuroprotective mechanisms have shown that SRG can reverse the suppressed expression of SIRT1, Beclin-1, and other autophagy markers in 6-OHDA-exposed cells. Thus, these enhanced the formation of autophagic vacuoles and autophagy activity. This protective effect can be blocked by pretreatment with wortmannin (an autophagosome formation blocker) and bafilomycin A1 (an autophagosome–lysosome fusion blocker). In addition, 6-OHDA increases the acetylation of Beclin-1, leading to its inactivation. SRG can induce the expression of SIRT1 and promote the deacetylation of Beclin-1. Finally, we found that SRG reduced the 6-OHDA-induced expression of miR-34a targeting *SIRT1*. The overexpression of miR-34a mimic abolishes the neuroprotective ability of SRG. In conclusion, SRG induces autophagy via partially regulating the miR-34a/SIRT1/Beclin-1 axis to prevent 6-OHDA-induced apoptosis and α-synuclein accumulation. SRG has the opportunity to be established as a candidate agent for the prevention and cure of PD.

## 1. Introduction

Parkinson’s disease (PD), which is common in the elderly, is the second most common neurodegenerative disease in the world after Alzheimer’s disease, caused by the progressive loss of dopaminergic (DA) neurons in the brain, resulting in insufficient dopamine [1]. It is related to aging (approximately 1–3% of the elderly over 60), various environmental factors (pesticides/chemicals/heavy metals and other toxic pollution sources), viral infection, and brain trauma. A small part is caused by genetic variation such as *SNCA*, *parkin*, and other mutations [2]. In addition, more than 70% of DA neuron loss will show clinical symptoms such as motor or psychocognitive behavior deficits in patients [3].

The establishment of Lewy bodies in neuronal cells is an important pathological marker of PD, composed of abnormal aggregation of proteins such as α-synuclein. Studies have shown that this abnormal aggregation can cause neuronal damage, sensitivity to stress, or apoptosis. In microglia, α-synuclein induces an inflammatory phenotype that causes neuroinflammation [4]. Autophagy is a tightly regulated conserved program accompanied by lysosomal machinery in cells. It can degrade and recycle misfolded proteins, other macromolecules, and damaged organelles. [5]. Genetic variants linked with impaired autophagy are also associated with the pathogenesis of PD [6]. Therefore, the PD therapeutic strategy can be achieved by upregulating the activity of the autophagy degradation system in clearing the abnormal aggregation of α-synuclein and damaged mitochondria.

Macroautophagy is the main pathway of autophagy, and its program includes four stages: the formation of autophagosomes, the fusion of autophagosomes and lysosomes, and the subsequent degradation and recycling of contents. First, the establishment of autophagosomal membranes is initiated. This is facilitated by a complex containing Beclin-1 and class III PI3K (Vps34). Next, the establishment of autophagosomes is induced. It is achieved via various Atg proteins and the alteration of LC3-I from LC3 to LC3-II molecules. Finally, autolysosomes are generated. The autophagosome is fused with the lysosome and uses acidic hydrolysis enzymes to degrade the waste content. Previous reports have shown that neurotoxins, including 6-OHDA, MPTP, and rotenone, can induce abnormality of autophagy in SH-SY5Y cells and DA neurons of mice [7,8,9]. Knockdown of Beclin-1 inhibits autophagic activity as well as removment of aggregated α-synuclein in PC12 [10]. The deficiency of Atg5 or Atg7 leads to an increase in cytoplasmic protein aggregates and damage in motor neurons of mouse models, showing the consequences of autophagy incapacitation [11]. Conversely, treatment with the autophagy activator rapamycin attenuates DA neuron loss in a mouse model of MPTP [12].

SIRT1 (Sirtuin-1) is an NAD-dependent deacetylase that removes acetyl groups from lysine residues within proteins in the presence of NAD^+^ [13]. The activity of SIRT1 is diminished by aging with a following augmented risk of developing various neuron degenerative diseases [14]. The enzymatic activity of SIRT1 has also been confirmed to be downregulated in PD patients, which may make the patients less protected against neuronal damage caused by various environmental factors [15]. Beclin-1, an essential protein for autophagy, can regulate autophagy by forming a complex with class III PI3K and other cofactors. Therefore, various modifications of Beclin-1 can regulate the activity of autophagy. The p300 can acetylate Beclin-1 to inhibit autophagosome maturation [16]. SIRT1 induces the deacetylation of Beclin-1 at K430 and K437 to promote autophagy [17]. Therapeutic applications of SIRT1 for its neuroprotective properties are gaining attention due to its powerful regulatory functions in autophagy, mitochondrial biogenesis, and mitophagy [18,19]. In SH-SY5Y cells, upregulation of SIRT1 expression can reduce the reactive oxygen species (ROS) production and promote autophagy to effectively counteract MPP^+^-induced apoptosis [20]. Similarly, increased SIRT1 activity in the 6-OHDA-exposed mouse model of PD ameliorated neuronal degeneration induced by mitochondrial damage in the substantia nigra [21].

microRNA (miRNA) is a non-coding, naturally occurring RNA molecule approximately 21–25 nucleotides in length. It regulates gene expression by partially complementing multiple messenger RNA (mRNA) molecules, causing translational repression, cleavage, and deadenylation of mRNA [22]. Studies have shown that SIRT1 is a target of mir-34a [23]. Mir-34a inhibits the expression of SIRT1 and affects cellular physiological phenomena such as senescence/aging and apoptosis [24,25].

**Figure 1:**
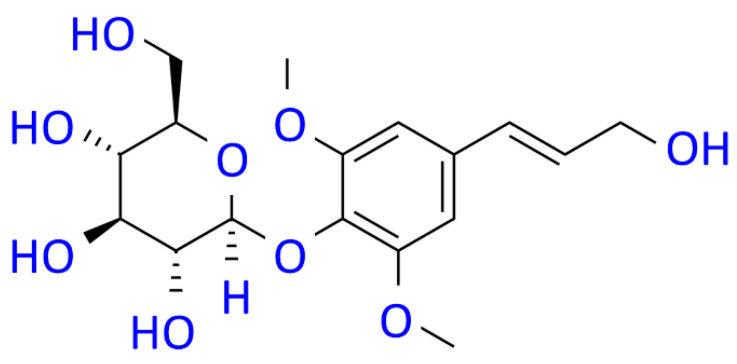
The molecular structure diagram of syringin (SRG) from *Acanthopanax senticosus*.

The biological activity of some phytocompounds is known to reduce PD risk [26]. Syringin (SRG) is a phenylpropanoid glucoside derived from the medicinal plant *Acanthopanax senticosus* and has various pharmacological functions, such as anti-inflammatory and antioxidant properties [27] (Figure 1). Studies have shown that SRG exhibits its anti-inflammatory and antioxidant cardioprotective effects through mediating SIRT1/PGC-1α and NRF2/HO-1 pathways in acute myocardial infarction models of cells and rats [27]. SRG can also alleviate the increase in ROS and apoptosis in Alzheimer’s disease cell models expressing Aβ_25–35_ [28]. In addition, studies on HeLa cells have revealed that SRG can induce autophagy [29]. In the mouse cardiac hypertrophy model, SRG can promote the expression of ATG5, ATG7, Beclin-1, and LC3 A/B [30]. In the present study, we evaluated the neuroprotective role of SRG in PD-related pathogenesis using 6-OHDA-exposed SH-SY5Y cells [31] and a *C. elegans* model [32]. The neurotoxin 6-hydroxydopamine (6-OHDA) can effectively induce pharmacological damage to DA neurons of animal models and SH-SY5Y cells. This is because it causes cells to yield excess ROS, thereby disrupting dopamine (enzymatic oxidation and autoxidation), the structural components of cells (DNA, lipids, and proteins), the function of mitochondria, autophagy, the ubiquitin–proteasome system (UPS), and finally promoting apoptosis [33]. In addition, the accumulation of α-synuclein, an important factor in the pathogenesis of PD, is known to impair autophagy and sensitize neurons to stresses such as ROS [34]. Here, we hypothesized that SRG mediates the regulation of autophagy. The results showed that SRG can promote neuroprotective mechanisms via activating autophagy partly through the miR-34a/SIRT1/Beclin-1 axis to prevent PD-associated molecular pathological features.

## 2. Materials and Methods

### 2.1. Chemicals, Handling, and Manipulation of SH-SY5Y Cells

Unless otherwise stated, chemicals and reagents were purchased from Sigma–Aldrich (St. Louis, MO, USA). Synthetic syringin (SRG, mol. wt. 372.4, 98% purity) was obtained from Rainbow Biotechnology Co., Ltd. (Shilin, Taipei, Taiwan) to prepare as a 1M (in DMSO) stock solution. The SH-SY5Y cell line was generously provided by Chia-Wen Tsai (China Medical University, Taichung, Taiwan) and was maintained according to the previously described culture method [35]. The medium and supplements were all purchased from Gibco, Thermo Fisher Scientific (Waltham, MA, USA). In SRG pretreatment, SH-SY5Y cells were cultured in a 6-well plate (2.5 × 10^6^/well), including a medium and the indicated concentrations of SRG. After 24 h, cells were added 100 μM of 6-OHDA to react for 12 or 18 h. In the autophagy inhibition experiments, 0.5 nM bafilomycin-A1 or 0.5 μM wortmannin was added 1 h before SRG pretreatment, respectively.

### 2.2. Viability Assay of SH-SY5Y Cells

We referred to previous experimental methods and steps for cell viability assays [35]. Succinctly, SH-SY5Y cells were seeded in a 96-well cell culture plate and treated as described in Section 2.1. Lastly, the CellTiter-Blue^®^ Reagent (Promega, Madison, WI, USA) was directly mixed with the culture medium and incubated at 37 °C for 2 h. Fluorescence signal intensity was detected using a spectrophotometer.

### 2.3. Detection of Mitochondrial Membrane Potential in SH-SY5Y Cells

We used the previous experimental approach to detect mitochondrial membrane potential [35]. In brief, SH-SY5HY cells were replaced with a fresh medium containing 1 μM 3,3′-dihexyloxacarbocyanine iodide (DiOC6). Green fluorescence was detected after 30 min using a fluorescence microscope, and the fluorescence signal intensity was quantified.

### 2.4. A Measure of DNA Fragmentation via TUNEL Assay in SH-SY5Y Cells

We conducted this assay according to the manufacturer’s direction (Invitrogen, Carlsbad, CA, USA) using the Click-iT™ Plus TUNEL Assay kit [35]. In short, fixed SH-SY5Y cells onto coverslips were preincubated with a Tdt reaction buffer for 10 min at 37 °C and then for a further 60 min at 37 °C with a Tdt reaction mixture. Next, the coverslips were detected with the Click-iT™ plus TUNEL reaction cocktail for 30 min at 37 °C in the dark. Finally, the cells were imaged via a fluorescence microscope.

### 2.5. Apoptosis Analysis Using Flow Cytometry of FITC-Annexin-V/PI on SH-SY5Y Cells

We implemented an apoptosis measure using the FITC Annexin-V Apoptosis Detection Kit I (BD Biosciences Pharmingen, San Diego, CA, USA) and flow cytometry according to the manufacturer’s introduction. In a nutshell, SH-SY5Y cells were suspended in a binding buffer, and FITC-Annexin-V/PI was added to stain for 15 min in the box. Next, cells were analyzed on a flow cytometer. The collection gate for a sample was 10,000 events.

### 2.6. Protein Expressing Analysis Using Western Blot on SH-SY5Y Cells

We performed Western blotting using previously established experimental methods [36]. SH-SY5Y cells were lysed, and the supernatant was collected. The total protein concentration was calculated using a Coomassie Plus protein assay kit (Pierce, Rockford, IL, USA). We took 50 µg of cell extract from each sample for electrophoresis analysis of the sodium dodecyl sulfate-polyacrylamide gel and then transferred the separated proteins to a polyvinylidene fluoride membrane. Next, the membrane was blocked and incubated with the primary antibody overnight at 4 °C. The next day, the membrane was washed, and HRP-conjugated secondary antibodies (PerkinElmer Inc., Boston, MA, USA) were added for 1 h at room temperature. Finally, specific protein signals were detected via the ECL substrate and chemiluminescent gel imager. PI3 kinase p100, Beclin-1, Atg 7, LC3, mTOR, phospho-mTOR, caspase 3, cleaved caspase 3, poly ADP ribose polymerase (PARP), cleaved PARP, SIRT1, β-tubulin, α-synuclein, and the β-actin antibody were all purchased from Cell Signaling Technology (Beverly, MA, USA).

### 2.7. Measuring ROS in SH-SY5Y Cells

We referred to previous experimental methods to determine ROS levels in SH-SY5Y cells [35]. Succinctly, SH-SY5Y cells were cultured in 96-well plates (lucifugal, 5 × 10^3^ cells/well), and then, 25 μM 2′,7′-dichlorodihydrofluorescein diacetate (H2DCFDA) was added for incubation for 30 min at 37 °C in the dark. Upon PBS washing of the cells, the fluorescence signal was recorded every 15 min for 150 min via a spectrophotometer.

### 2.8. Staining of Acridine Orange in SH-SY5Y Cells

We used the previous experimental approach to detect acidic vesicular organelles [37]. Cells were incubated with a fresh medium, and acidic vesicular organelles were detected via acridine orange staining.

### 2.9. Measuring Autophagic Activity in SH-SY5Y Cells

The autophagic activity of the SH-SY5Y cells was determined using the Autophagy Assay Kit (Sigma–Aldrich) based on the product’s manual. Cells in 96-well plates (1 × 10^4^ cells/well) were washed, and a detection solution was added. After 1 h, cells were washed and measured via a spectrophotometer.

### 2.10. Immunoprecipitation Assay of Acetylation of Beclin-1 in SH-SY5Y Cells

We used the previous experimental approach for the immunoprecipitation assay [17]. In brief, cell lysates were incubated with a Beclin-1 antibody overnight at 4 °C, followed by the addition of protein A-sepharose beads for 4 h. Next, immunoprecipitated complexes were collected via centrifugation, and the pellets were washed twice with an immunoprecipitation buffer. Subsequently, the acetylation of Beclin-1 was determined via Western blotting using the Acet K monoclonal antibody (Cell Signaling Technology).

### 2.11. Measurement of Human MiR-34a Expression via RT-qPCR in SH-SY5Y Cells

According to the manufacturer’s instructions, miRNAs were isolated using the High Pure miRNA Isolation Kit (Roche Diagnostics, GmbH, Mannheim, Germany), and the concentration of the extracted RNA was calculated using an ultraviolet spectrophotometer. miRNA was reverse transcribed using the MystiCq microRNA cDNA Synthesis Mix kit (Sigma–Aldrich). Finally, we used the MystiCq microRNA qPCR Assay System (Sigma–Aldrich) and the ABI StepOnePlus system (Applied Biosystems, Inc., Foster City, CA, USA) to detect the miR-34a expression. The primer sequences were 5′-GGCAGTGTCTTAGCTGG-3′ (forward) and 5′-GAACATGTCTGCGTATCTC-3′ (reverse). Amplification was performed under the following protocol: pre-degeneration at 94 °C for 10 min, at 94 °C for 20 s, at 68 °C for 30 s, and at 72 °C for 30 s, respectively; 35 cycles in total. U6 was taken as a reference gene. The expression quantity was calculated via the 2^−ΔΔCq^ method.

### 2.12. Transient Transfection of Human MiR-34a Inhibitors and Mimics in SH-SY5Y Cells

The miR-34a mimic (mature sequence: 5′-CAAUCAGCAAGUAUACUGCCCU-3′), miRNA-negative control, miR-34a inhibitor (mature sequence: 5′-UGGCAGUGUCUUAGCUGGUUGU-3′), and inhibitor negative control were obtained from Sigma–Aldrich. According to the product’s introduction, we used Lipofectamine 2000 (Invitrogen) to transiently transfect SH-SY5Y cells with the inhibitor, mimics, and their negative controls, respectively. The initial concentration was 50 µM. After 12 h of transfection, the cells were treated as described in Section 2.1.

### 2.13. C. elegans Strains and Maintenance/Synchronization of Worms

The Caenorhabditis Genetics Center (University of Minnesota, Saint Paul, MN, USA) offered our following biological resources: wild-type Bristol N2 *C. elegans*, transgenic BZ555 strain (Pdat-1::GFP), transgenic N5901 strain (Punc-54::α-Syn::YFP), transgenic DA2123 strain (lgg-1p::GFP::lgg-1), transgenic VC199 strain [sir2.1 (ok434)], and *E. coli* strain OP50. The general cultivation and synchronization of worms was implemented using an earlier-described protocol [35].

### 2.14. Determining the Optimal Treatment Concentration of SRG for Worms via Food Clearance Assay

We followed the previous experimental procedure for the food clearance assay [38]. Simply put, OP50 *E. coli* cultured overnight was added to an S medium, including a serial dilution of SRG. Then, the S medium containing SRG and OP50 was loaded into a 96-well plate with twenty L1-stage worms. Lastly, the OD value of the culture was detected using a spectrophotometer every day during the six-day culture period.

### 2.15. Pretreatment of SRG and Exposure to 6-OHDA in Worms

We used an SRG/OP50/NGM plate to culture worms of the L1 stage until the L3 stage, and then, worms were soaked in a 50 mM 6-OHDA solution containing 10 mM ascorbic acid for 1 h. Lastly, worms were washed and removed to an SRG/OP50/NGM/FUDR plate for growth. After 3 days, the worms were used for the experiments in this study unless otherwise indicated.

### 2.16. Analysis of Degeneration of 6-OHDA-Induced DA Neurons in BZ555 Worms

BZ555 worms were treated as described in Section 2.16. Then, the worms were washed, anesthetized, and placed on 2% agar pad-contained slides. Finally, the worms were covered with a coverslip and observed via a fluorescence microscope. The fluorescence images of DA neurons in worms’ heads (three pairs) were analyzed. In addition, we visually calculated the degeneration of DA neurons in worms. DA neurons in worms were considered to be degenerated if the dendrites were bubbling or disappearing.

### 2.17. Assessing DA Neuron Function via Food Sensitivity Behavioral Test in N2 Worms

N2 worms were treated as described in Section 2.16. First, we incubated OP50 on a 9 cm NGM plate with an outer diameter of 8 cm and an inner diameter of 1 cm overnight. After that, the washed worms were dripped in the center of the plate. About 5 min later, the number of S-shaped movements of the worm within 20 s on the lawn without and with OP50 was measured. Each worm was counted three times. Fifty worms were evaluated per group.

### 2.18. The Analysis of Lifespans in N2 Worms

In the lifespan assessment of worms, we removed N2 worms of the L3 stage to NGM/OP50/FUDR/plates with or without SRG for culture. Then, worms were removed to fresh plates every 3 days until 50 worms per group died. Using a dissecting microscope, we counted the number of surviving worms daily. Dead worms were defined as unresponsive to repeated contact with worm pickers. Survival curves were obtained for analysis based on the Kaplan–Meier method and SPSS software (version 25, IBM, Armonk, NY, USA).

### 2.19. Measuring of α-Synuclein Accumulation in Muscle Cells of NL5901 Worms

NL5901 worms of the L3 stage were cultured on NGM/OP50/FUDR plates with or without SRG for 3 days. In muscle cells of the worm body, the accumulation of YFP-fused α-synuclein was measured via fluorescence microscopy.

### 2.20. The Analysis of α-Synuclein Protein Expression in NL5901 Worms

Snap-frozen worms in Section 2.19 were extracted total protein via Fastprep24 (MP Biomedicals LLC, Solon, OH, USA) with protease inhibitors/PBS under liquid nitrogen. Western blotting was performed according to Section 2.6.

### 2.21. Measuring Autophagic Activity Using DA2123 Worms

DA2123 worms had a GFP-fused LGG-1 gene, including the original promoter sequence. We used these worms to perform in vivo autophagy activity analysis. Handling of DA2123 worms was the same as in Section 2.20. We calculated the LGG-1::GFP positive puncta number in the lateral epidermal seam cells (at least ten cells). At least fifty worms were evaluated per group.

### 2.22. Detecting the ROS Level in N2 Worms

We referred to a previous operation method to measure ROS levels in worms [35]. Thirty worms from each group were washed and transferred to a 96-well plate in PBS. Next, we mixed worms with H2DCFDA and detected fluorescence signals using a spectrophotometer. The well was measured every 15 min over a 150 min reaction time.

### 2.23. Extraction of Total RNA and RT-qPCR in Worms

We extracted total RNA from frozen worms using glass beads in a TRIzol reagent (Invitrogen) with vigorous shaking as the manufacturer recommended. Next, we used the SuperScript One-Step RT-PCR kit (Invitrogen), SYBR Green I Master kit (Roche Diagnostics, Indianapolis, IN, USA), and ABI StepOnePlus system (Applied Biosystems) for RT-qPCR analysis to quantify target gene expression. Finally, relative expression levels were calculated using the 2^−ΔΔCT^ method. Gene expression data were normalized by the control gene *act-2*. The primer sequences for *sir-2.1* were 5′-TGGCTGACGATTCGATGGAT-3′ (forward) and 5′-ATGAGCAGAAATCGCGACAC-3′ (reverse). For *act-2*, they were 5′-CCCACTCAATCCAAAGGCTA-3′ (forward) and 5′-GGGACTGTGTGGGTAACACC-3′ (reverse).

### 2.24. Statistical Analysis in this Study

Data were analyzed using commercial statistical SPSS software (version 25, SAS Institute Inc., Cary, NC, USA). Each experiment was implemented at least three times. Results were presented as means ± standard deviations (SDs). Statistical difference was calculated via one-way ANOVA with Tukey’s post hoc test. The *p*-values < 0.05 were regarded as statistically significant.

## 3. Results

### 3.1. Syringin (SRG) Improves Apoptosis and Exhibits Neuroprotective Activity in SH-SY5Y Cells Exposed to 6-OHDA

**Figure 2:**
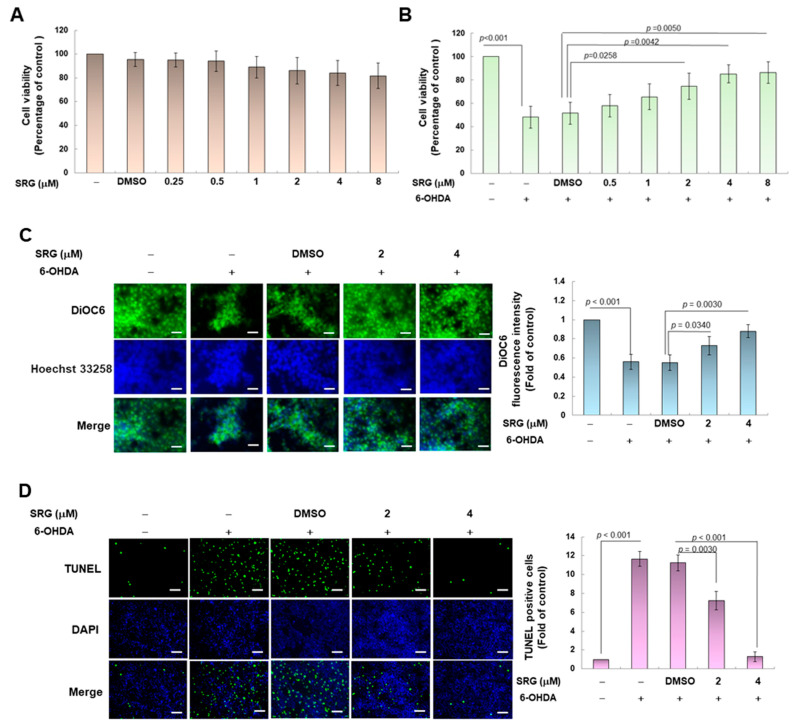

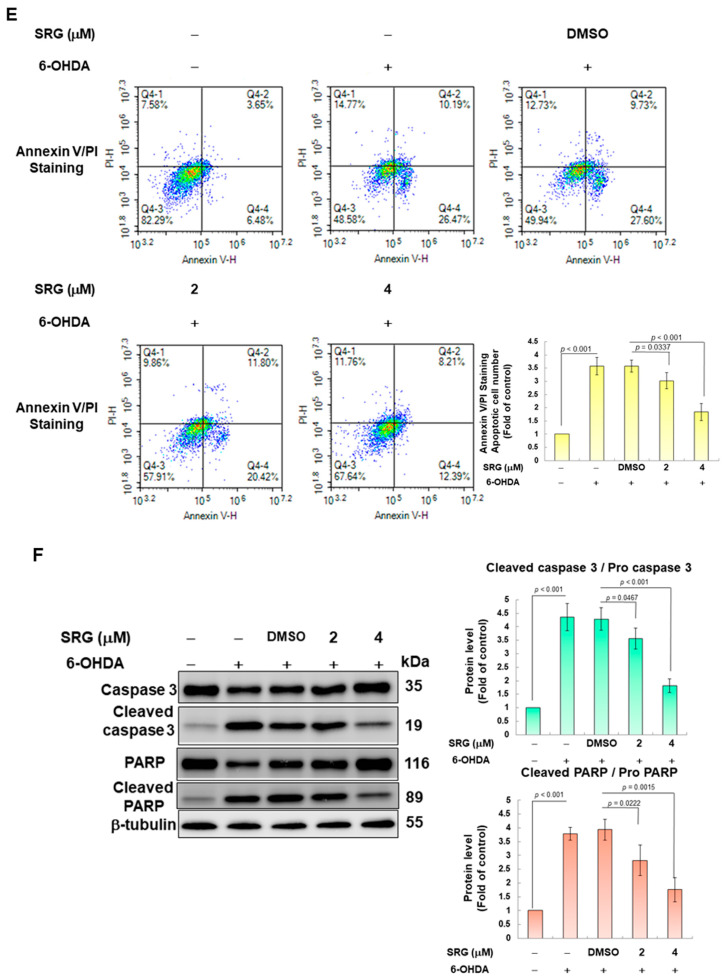
SRG prevents the apoptosis of SH-SY5Y cells damaged via 6-OHDA. (**A**) SH-SY5Y cells were treated with serially diluted SRG. The percentage of viable cells after 24 h was determined using the CellTiter Blue cell viability assay. (**B**) Cell viability was analyzed following exposure of (**A**)-treated cells to 100 μM 6-OHDA for 18 h. (**C**) DiOC6 staining was used to analyze changes in the mitochondrial membrane potential (MMP) of cells treated with (**B**) under a fluorescence microscope (scale bar = 50 µm). The fluorescence intensity of the image was estimated via ImageJ software (version 1.53). (**D**) The proportion of cells with apoptosis-related DNA fragmentation was determined via fluorescence microscopy (scale bar = 100 µm) using TUNEL staining. (**E**) Cells treated with (**B**) were analyzed via flow cytometry to determine the number of apoptotic cell populations presented via Annexin V-FITC binding and propidium iodide (PI) staining. (**F**) Changes in levels of apoptosis-related proteins in cells treated with (**B**) were analyzed via Western blotting. β-tubulin was used to normalize the level of total protein in each group.

In this study, we first employed the SH-SY5Y model damaged via 6-OHDA neurotoxins to analyze the neuroprotective ability of SRG. In order to obtain a suitable nontoxic treatment dose of SRG, we treated cells with serially diluted SRG and performed the CellTiter Blue cell viability assay reflecting the metabolic activity of living cells 24 h later. We found that treating cells at concentrations below 8 μM SRG did not affect cell survival (Figure 2A). Next, SRG-pretreated cells were exposed to 6-OHDA (100 μM). After 18 h of treatment, the results showed that SRG pretreatment (2 to 4 μM) dose-dependently reduced cell death (Figure 2B). Compared with the 6-OHDA (DMSO) group, pretreatment with 4 μM SRG increased the cell survival rate by 1.6-fold (*p* = 0.0042). Therefore, we used SRG treatment concentrations of 2 and 4 μM as benchmarks for subsequent studies.

Studies have shown that the toxicity of 6-OHDA is related to the induction of apoptosis activity [39]. Therefore, we evaluated the protective utility of SRG in the prevention of apoptosis associated with 6-OHDA. A decline in mitochondrial membrane potential (MMP) reflects the onset of apoptosis [40]. We stained with a fluorescent dye that reflects the MMP and showed that 6-OHDA inhibited the MMP by 44.0% (*p* < 0.001) compared to the control group (Figure 2C). Compared with the 6-OHDA (DMSO) group, SRG pretreatment (4 μM) increased MMP by 1.6-fold (*p* < 0.001, Figure 2C). Moreover, we used a TUNEL assay to determine the degree of DNA fragmentation related to apoptosis. We found that DNA fragmentation in cells increased up to 11.7-fold (*p* < 0.001) upon exposure to 6-OHDA compared to the control group (Figure 2D). While the SRG pretreatment group (4 μM) was compared with the 6-OHDA (DMSO) group, the cellular DNA fragmentation was reduced by 88.4% (*p* < 0.001) (Figure 2D). Furthermore, the situation of apoptosis was verified via double staining of annexin V-FITC and propidium iodide (PI) combined with flow cytometry via the signal strength of phosphatidylserine (PS) on the cell surface and loss of cell membrane permeability. We found a combined 3.6-fold increase in PS and PS/PI cell populations in the 6-OHDA group compared to the control group (*p* < 0.001) (Figure 2E). However, pretreatment with SRG (4 μM) reduced the PS and PS/PI cell populations by 50.0% (*p* < 0.001) compared with the 6-OHDA (DMSO) group (Figure 2E).

In addition, our analysis of the expression and maturation of apoptosis-related proteins is also in line with the above observations. In SH-SY5Y cells exposed to 6-OHDA, the ratio of cleaved caspase 3 to procaspase 3 increased by 4.4-fold (*p* < 0.001), similarly, the ratio of cleaved PARP to pro PARP increased by 3.8-fold (*p* < 0.001, Figure 2F). Under SRG (4 μM) pretreatment, the ratio of cleaved caspase 3 to pro caspase 3 was reduced by 58.1% compared with the 6-OHDA (DMSO) group (*p* < 0.001). The ratio of cleaved PARP to pro PARP was reduced by 53.8% (*p* = 0.0015) (Figure 2F). The above data show that SRG can concentration-dependently reverse the effect of 6-OHDA on SH-SY5Y cell apoptosis.

### 3.2. SRG Reduces ROS Production Induced via 6-OHDA in SH-SY5Y Cells via Activating Autophagy

**Figure 3:**
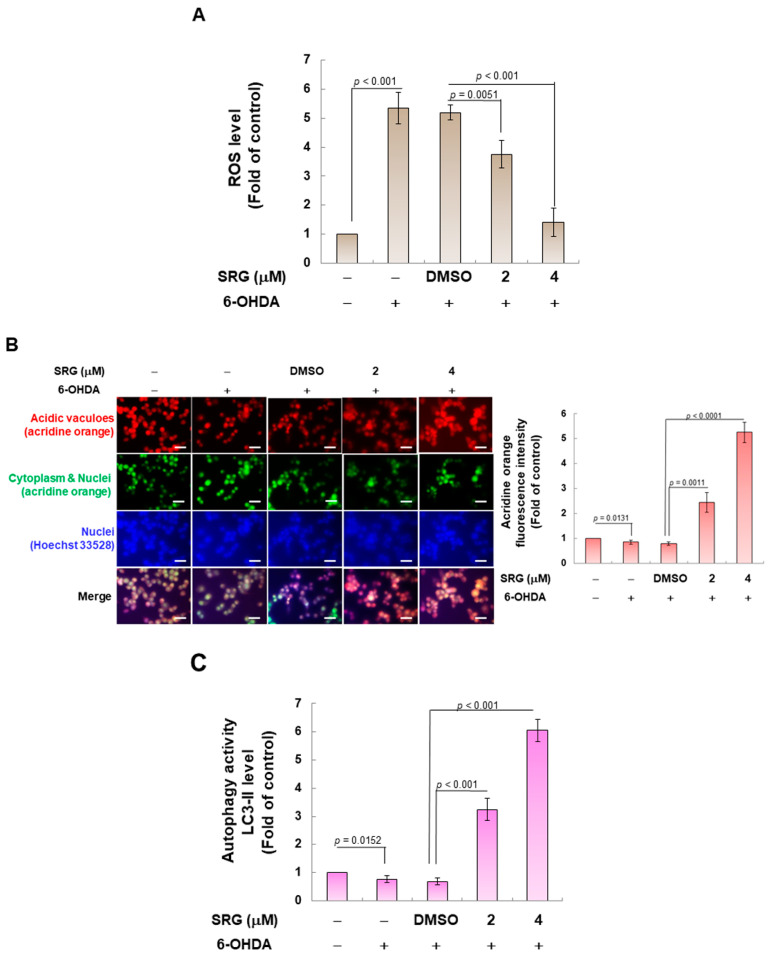

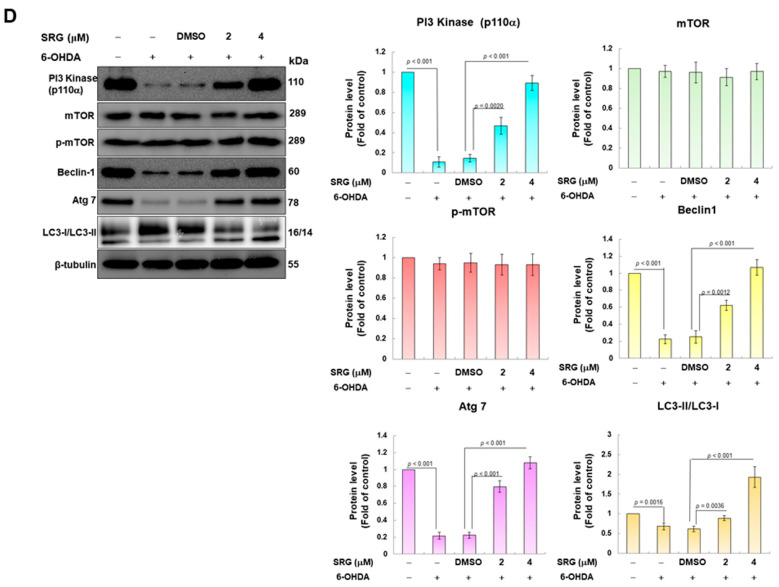
SRG pretreatment decreased the production of cellular ROS and increased the autophagy in the 6-OHDA-exposed SH-SY5Y cell model. After 24 h of SRG pretreatment, cells were exposed to 100 μM 6-OHDA for an additional 18 h. (**A**) The ROS production in cells was analyzed via H2DCFDA probe and spectrophotometer. (**B**) The acidic vacuoles level in the cells was stained with acridine orange and then observed using a fluorescence microscope (scale bar = 50 µm). Fluorescence intensity was quantified via ImageJ software. (**C**) Autophagy activity in the cells was analyzed via LC3-II staining and spectrophotometer. Signal intensity was quantified via ImageJ. (**D)** After SRG pretreatment for 24 h, SH-SY5Y cells were exposed to 100 μM 6-OHDA for 12 h, and finally, the expression of autophagy-related genes was analyzed via Western blotting. The histogram is the result of quantitative analysis of the measured signal using ImageJ. β-tubulin was used to normalize the level of total protein in each group.

The neurotoxicity of 6-OHDA is mainly due to the massive production of ROS caused via mitochondrial damage of DA neurons. Here, we assessed the antioxidant capacity of SRG using the fluorescent dye H2DCFDA. The results showed that compared with the control group, the production of ROS in the 6-OHDA group increased by 5.4-fold (*p* < 0.001, Figure 3A). The SRG (4 μM) pretreatment group, compared with the 6-OHDA (DMSO) group, reduced the level of intracellular ROS by 73.1% (*p* < 0.001, Figure 3A).

Previous studies have shown that increased autophagy activity helps cells resist ROS toxicity caused via mitochondrial damage [41]. Because one of the characteristic components of autophagy is the production of autophagosomes [42]. First, we determined the autolysosome formation using simple acridine orange staining. As shown in Figure 3B, 6-OHDA treatment slightly reduced the establishment of autolysosome (*p* = 0.0131). SRG treatment concentration-dependently increased the production of autolysosomes. Compared with the 6-OHDA (DMSO) group, SRG (4 μM) increased autolysosome formation up to 6.6-fold (*p* < 0.001, Figure 3B). We also determined the autophagic activity using LC3-II-based ELISA assays. The data revealed that 6-OHDA exposure slightly reduced the accumulation of LC3-II (*p* = 0.0152, Figure 3C). However, compared with the 6-OHDA (DMSO) group, SRG (4 mM) increased the accumulation of LC3-II by 8.9-fold (*p* < 0.001, Figure 3C).

Next, we used Western blotting to observe the changes in the level of proteins associated with autophagy. Class III PI3K signaling upregulates the autophagic pathway. The protein expression of PI3 kinase p100 (*p* < 0.001), Beclin-1 (*p* < 0.001), and Atg7 (*p* < 0.001) was decreased under 6-OHDA exposure. Likewise, the ratio of LC3-II/LC3-I (*p* = 0.0016) was also significantly diminished (Figure 3D). Compared with the 6-OHDA (DMSO) group, SRG pretreatment significantly enhanced the protein expression of PI3 kinase p100, Beclin-1, Atg7, and the ratio of LC3-II/LC3-I in a concentration-dependent manner (Figure 3D). The protein expression of PI3 kinase p100, Beclin-1, and Atg7 and the ratio of LC3-II/LC3-I increased by 6.1-fold (*p* < 0.001), 4.3-fold (*p* < 0.001), 4.8-fold (*p* < 0.001), and 3.1-fold (*p* < 0.001) under SRG pretreatment (4 μM), respectively. Class I PI3K signaling (PI3K/Akt pathway) is known to down-regulate the induction of autophagy via mTOR, but in the present study, we found that SRG did not affect the activation (phosphorylation) of mTOR in SH-SY5Y cells (Figure 3D). Therefore, the ability of SRG to induce autophagy may be caused by class III PI3K signaling independent of the class I PI3K/mTOR pathway.

### 3.3. The Ability of SRG to Reverse the Autophagy Dysfunction and Apoptosis Caused by 6-OHDA Was Abolished via Wortmannin and Bafilomycin A1 Treatment

**Figure 4:**
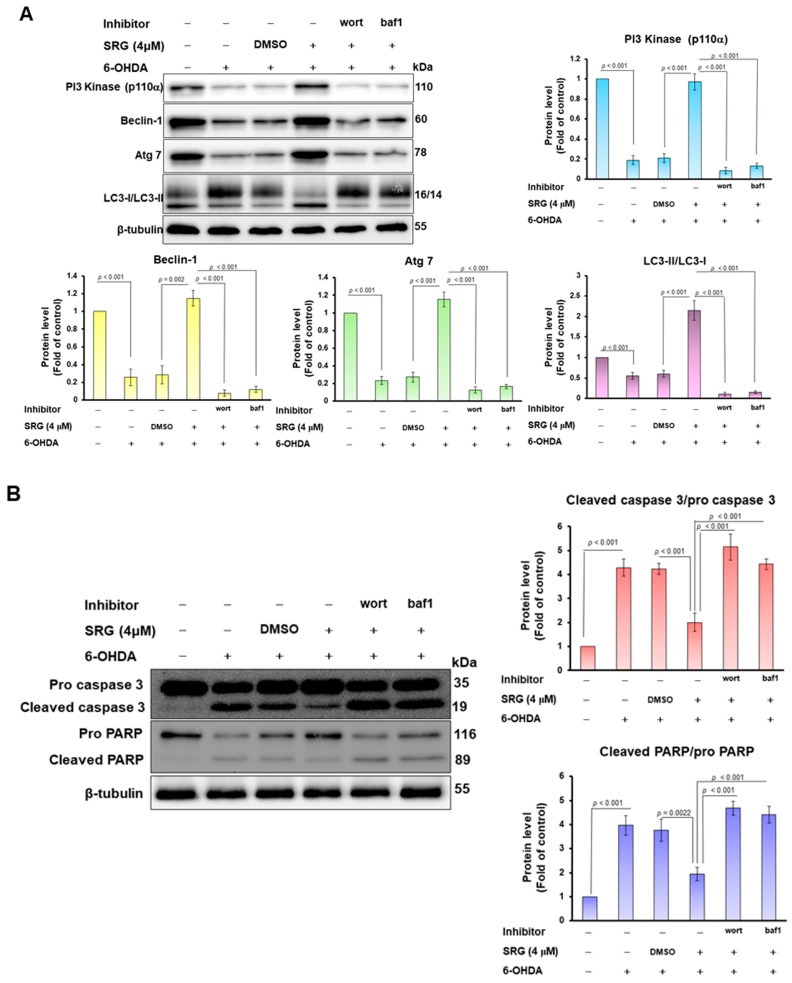
Treatment with wortmannin and bafilomycin A1 abolished the character of SRG to reverse the protein expression associated with the down-regulation of autophagy and enhancement of apoptosis caused by 6-OHDA exposure. SH-SY5Y cells were pretreated with SRG for 24 h after adding 0.5 μM wortmannin (wort) or 0.5 nM bafilomycin A1 (baf-1) for 1 h and then incubated with 6-OHDA for an additional 12 h. (**A**) The protein level of PI3 kinase p100, Beclin-1, Atg7, and the ratio of LC3-II/LC3-I were measured via Western blotting. (**B**) The level of apoptosis-related proteins was detected via Western blotting. β-tubulin was used as a loading control. Alterations in protein levels were determined via ImageJ software.

To confirm that the property of SRG to reverse 6-OHDA cytotoxicity is connected with autophagic activation, we used wortmannin (an early blocker of autophagosome formation) and bafilomycin A1 (autophagosome–lysosome fusion blockers). The result showed that pretreatment of cells with these blockers reversed the ability of SRG to upregulate PI3 kinase p100, Beclin-1, Atg7 protein levels, and the LC3-II/LC3-I ratio against 6-OHDA exposure (Figure 4A). Next, we used wortmannin and bafilomycin A1 to confirm whether the anti-apoptotic effect of SRG on 6-OHDA toxicity was related to the activation of autophagy. The results revealed that treatment of cells with these two blockers abolished the ability of SRG to reverse the upregulation of the apoptosis-associated caspase 3/cleaved caspase 3 and PARP/cleaved PARP ratio upon 6-OHDA exposure (Figure 4B).

### 3.4. SRG Restores 6-OHDA-Induced Downregulation of SIRT1 Protein Expression and Promotes Deacetylation of Beclin-1

**Figure 5:**
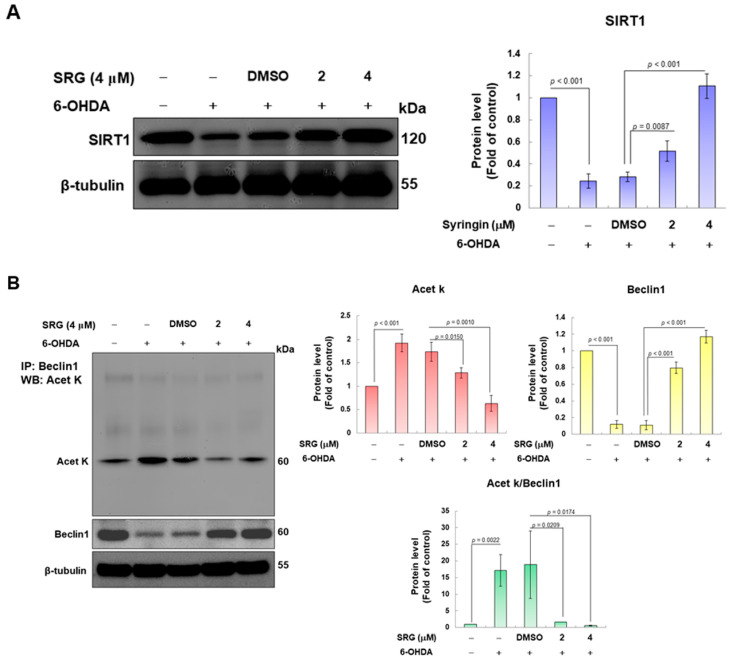
The 6-OHDA caused the downregulation of the SIRT1 protein expression, and the acetylation of Beclin-1 was reversed via SRG pretreatment in SH-SY5Y cells. (**A**) The expression of the SIRT1 protein in SH-SY5Y cells was analyzed via Western blotting. β-tubulin was used as a loading control. The protein expression was estimated via ImageJ software. (**B**) The lysate of SH-SY5Y cells was immunoprecipitated with a Beclin-1 antibody and then analyzed via Western blotting using an anti-acetylated lysine residue antibody. In addition, β-tubulin was used as the lysate input control. Consistent Beclin-1 expression in the lysate of each sample was used as the benchmark for determining the degree of acetylation.

The SIRT1 protein has been linked to the activation of autophagy. Its ability to deacetylate can affect the activity of many autophagy-associated proteins, including Beclin-1 [17]. Therefore, we wanted to explore whether SRG affects SIRT1 protein expression. Western blotting analysis showed that the expression of the SIRT1 protein was significantly reduced in 6-OHDA-exposed cells (*p* < 0.001, Figure 5A). Nevertheless, SRG concentration-dependently increased the suppressed SIRT1 level in 6-OHDA-exposed cells (Figure 5A). Under SRG (4 μM) pretreatment, the expression of SIRT1 increased 3.9-fold compared with the 6-OHDA (DMSO) group (*p* < 0.001, Figure 5A). Next, we analyzed the degree of acetylation of Beclin-1 using the co-immunoprecipitation of a Beclin-1 antibody combined with Western blotting analysis using the specific antibody for acetylation of lysine residues. We adjusted the expression of Beclin-1 in each sample to make it consistent. Based on this, each sample’s degree of acetylation of Beclin1 was compared. Results displayed that 6-OHDA exposure augmented the acetylation of intracellular Beclin-1 by 17.2-fold (*p* < 0.001, Figure 5B). SRG could concentration-dependently reduce the acetylation of Beclin-1 induced via 6-OHDA. Pretreatment at 4 μM SRG reduced the acetylation of Beclin-1 by 97.2% compared to the 6-OHDA (DMSO) group (*p* = 0.0174, Figure 5B). Thus, SRG potentiates SIRT1-dependent deacetylation of Beclin-1 in 6-OHDA-exposed cells.

### 3.5. The 6-OHDA-Induced Upregulation of Endogenous MiR-34a Targeting SIRT1 Was Reversed via SRG Pretreatment

**Figure 6:**
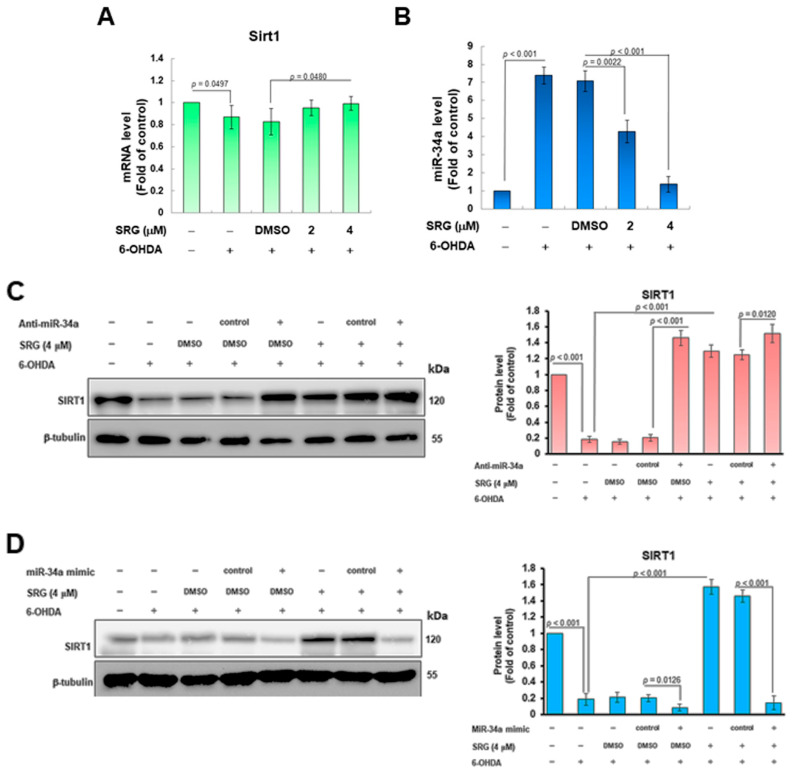
SRG reduces the upregulation of miR-34a expression induced via 6-OHDA exposure to restore SIRT1 expression in SH-SY5Y cells. (**A**) RT-qPCR analysis of *SIRT1* mRNA expression in SH-SY5Y cells of each group. (**B**) RT-qPCR analysis of miR-34a levels in SH-SY5Y cells of each group. (**C**,**D**) The role of miR-34a in the SRG-enhanced SIRT1 pathway was confirmed using (**C**) anti-miR-34a (miR-34a inhibitor) and (**D**) miR-34a mimics. The level of the SIRT1 protein in SH-SY5Y cells of each group was analyzed via Western blotting. β-tubulin was used as an internal loading control, and the signal was quantified via ImageJ software.

To confirm the possible mechanism of SRG-induced SIRT1 activity, we first observed the expression of SIRT1 mRNA using RT-qPCR. Quantitative results showed that 6-OHDA exposure slightly reduced the expression of SIRT1 mRNA in SH-SY5Y cells (*p* = 0.0497, Figure 6A). After treatment with SRG (4 μM), the level of SIRT1 mRNA was slightly increased compared with the 6-OHDA (DMSO) group (*p* = 0.0480, Figure 6A). Interestingly, this result does not match the observation of significant differences in the level of SIRT1 protein expression. Various endogenous miRNAs are known to regulate SIRT1 expression mainly by blocking the translation. This way has less impact on the level of SIRT1 mRNA. We assumed that SRGs regulate the expression of specific SIRT1-targeted miRNAs. We selected several of these miRNAs for expression analysis. The RT-qPCR indicated that the expression of miR-34a was significantly amplified after 6-OHDA exposure compared with the control group (*p* < 0.001, Figure 6B). SRG pretreatment could decrease the expression of miR-34a in a concentration-dependent manner (Figure 6B). After pretreatment with SRG (4 μM), compared with the 6-OHDA (DMSO) group, the expression of miR-34a decreased by 81% (*p* < 0.001, Figure 6B), which is more consistent with the increased expression of the SIRT1 protein (Figure 6B). To confirm the role of miR-34a in the SRG-activated SRIT1 pathway, we used the transfection of anti-miR-34a (miR-34a inhibitor). The results showed that SIRT1 protein expression was significantly increased in the anti-miR-34a group compared with the anti-miR control group under 6-OHDA exposure (*p* < 0.001, Figure 6C). At this point, however, SIRT1 expression could no longer be enhanced via pretreatment with SRG (Figure 6C). Furthermore, we found that transfection of the miR-34a mimic further suppressed SIRT1 expression in 6OHDA-exposed cells (*p* < 0.001, Figure 6D) and abolished the effect of SRG in promoting the increase of SIRT1 levels in this setting (Figure 6D). The above data suggest that miR-34a is a more upstream pathway in SRG-mediated SIRT1 activity.

### 3.6. The 6-OHDA-Induced Degeneration of DA Neurons in Caenorhabditis Elegans Can Be Reversed via SRG Pretreatment

**Figure 7:**
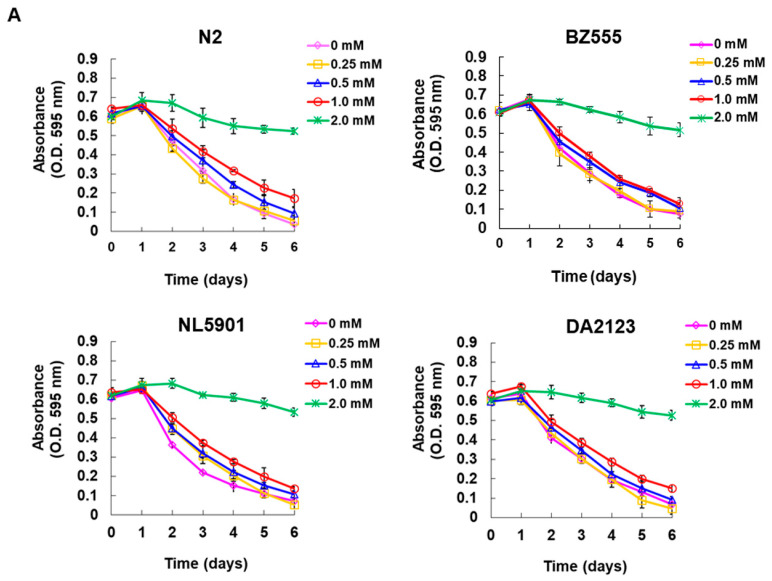

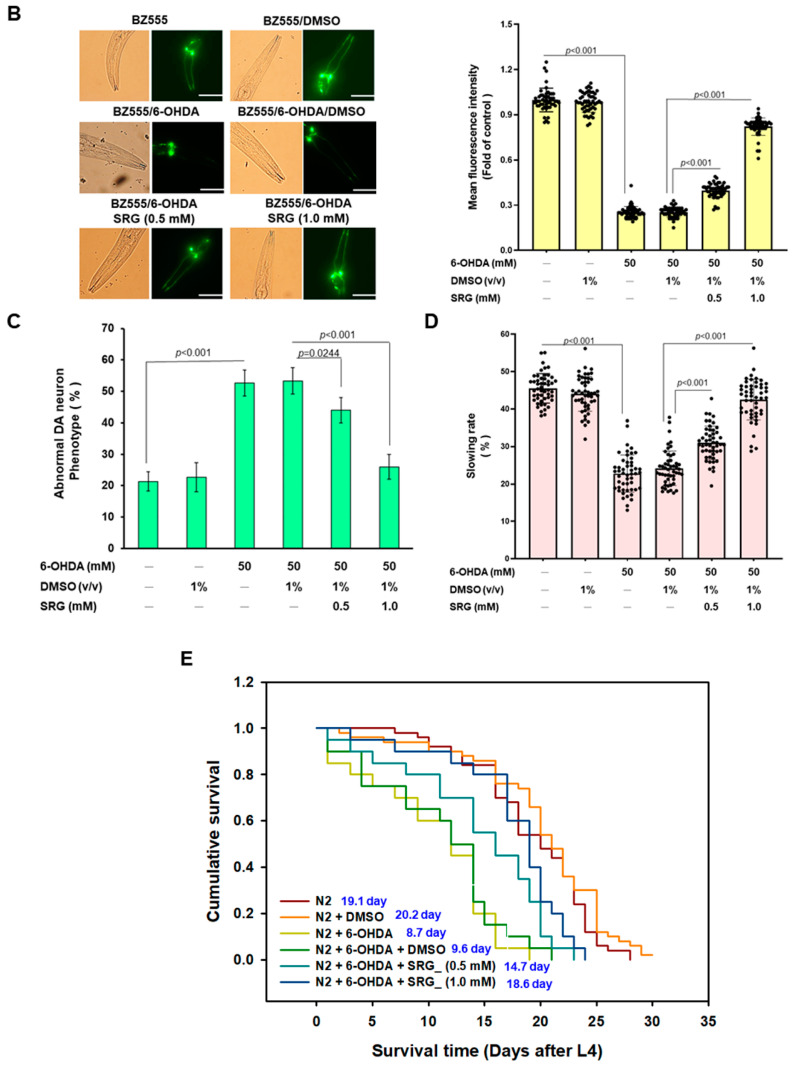
The 6-OHDA neurotoxicity-induced deficits in DA neuron integrity, food-sensitive behavior, and lifespan in *C. elegans* were ameliorated via SRG pretreatment. (**A**) A food clearance test was used to obtain an appropriate dose of SRG for treating worms. The L1-stage worms (approximately 20) of the used strains were placed in 96-well plates containing *Escherichia coli* OP50 (OD A_595_ = 0.6) and different concentrations of SRG in an S medium for 6 days (20 °C). The OD value of each well was recorded every day. (**B**) Fluorescent images of GFP-labeled DA neurons in the head of BZ555 worms from each group. Fluorescence signals were calculated using ImageJ software. Scale bar = 50 µm. (**C**) The phenotype deficit of DA neurons in (**B**) was visually assessed and expressed as a percentage. If the axonal fluorescence signal was punctated or disappeared, it was considered degeneration. (**D**) The effect of SRG on the restoration of the DA neuronal function in 6-OHDA-exposed N2 worms was evaluated in a food-sensitivity behavioral assay. The slowing rate is expressed as the percentage reduction in the frequency of the S-shaped movement (20 s) of the worms moving from the empty lawn to the bacterial lawn. Fifty worms per group were counted. (**E**) The lifespan of N2 worms was assessed via cumulative survival curves. The worms in each group were replaced with fresh plates every three days, and the number of surviving worms was calculated every day. Fifty worms per group were counted.

Since the utility of SRG needs to be further evaluated in animal models, we adopted the widely used PD model of *C. elegans* (worms). First, to confirm the acceptable therapeutic dose of SRG treatment in this model, we used a food clearance test to observe the effect of different SRG doses on the physiologically relevant food intake of worms. The result showed that when the S medium was added up to 2 mM SRG, the food clearance curves of each strain of worms were significantly slowed down compared to the untreated group (Figure 7A). These results show that SRG at 2 mM is toxic to the normal physiological functions of the worms. Since SRG concentrations below 1 mM did not significantly alter the food clearance curve (Figure 7A), we treated worms with up to 1 mM SRG in the following experiments.

To assess the protective efficacy of SRG in worms against the 6-OHDA neurotoxin-induced degeneration of DA neurons, we performed assays using BZ555 worms expressing uniform GFP in DA neurons. Fluorescence microscopy observations showed that 6-OHDA exposure mainly caused damage and degeneration (integrity destruction) of DA neurons in the head, resulting in a significant decrease in fluorescence intensity (Figure 7B). Compared with the control group, the mean fluorescence intensity was diminished by 74.7% in the 6-OHDA group (*p* < 0.001, Figure 7B). However, fluorescence signals in 6-OHDA-exposed worms were dose-dependently restored via SRG pretreatment (Figure 7B). Pretreatment with 1 mM SRG increased the fluorescence intensity downregulated via 6-OHDA by 2.5-fold compared to the 6-OHDA (DMSO) group (*p* < 0.001, Figure 7B). Furthermore, visual observation of 6-OHDA-exposed worms showed that the ratio of abnormal phenotypes of DA neurons was significantly higher than that of unexposed worms by 3.9-fold (*p* < 0.001, Figure 7C). SRG pretreatment reversed this phenomenon in a dose-dependent manner. Pretreatment with SRG (1 mM) significantly reduced the phenotype of DA neuron degeneration in 6-OHDA-exposed worms by 51.2% (*p* < 0.001, Figure 7C) compared to the 6-OHDA (DMSO) group.

### 3.7. The Loss of Dopamine-Mediated Food-Sensitive Behavior in Worms Induced via 6-OHDA Exposure Can Be Reversed via SRG Pretreatment

Food-sensitive behavior in *C. elegans* is mediated via dopamine in DA neurons [43]. When worms touch food, they reduce the frequency of their S-shaped body bending (movement speed) to improve their feeding efficiency. Wild-type N2 worms in this study showed a 45.1% reduction in bending frequency (slowing rate) after exposure to bacterial lawns compared with empty lawns (Figure 7D). In 6-OHDA-exposed worms, the slowing rate was significantly reduced by 50.6% compared with the control group (*p* < 0.001, Figure 7D). SRG could restore this slowing rate in a dose-dependent manner. The slowing rate increased 1.8-fold in the 1 mM SRG group compared with the 6-OHDA (DMSO) group (*p* < 0.001, Figure 7D). The above results showed that the function of DA neurons damaged via 6-OHDA could be reversed via SRG pretreatment.

### 3.8. The Shortened Lifespan of Worms Due to 6-OHDA Toxicity Can Be Restored via SRG Pretreatment

The neurotoxicity of 6-OHDA shortens the lifespan of *C. elegans* in studies [44]. Here, we wondered whether SRG could improve worm lifespan shortened via 6-OHDA toxicity. The results showed that the average survival days of the 6-OHDA-exposed worms were 8.7 ± 2.8 days, while the average survival days of the control worms were 19.1 ± 1.7 days (Figure 7E). Notably, the average survival days in the 1 mM SRG group were significantly prolonged to 18.6 ± 2.2 days compared with the 6-OHDA (DMSO) group (*p* < 0.001 (Figure 7E). Thus, SRG could reverse the shortened lifespan induced via neurotoxin 6-OHDA in worms.

### 3.9. The α-Synuclein Accumulating in Muscle Cells of Worms Can Be Alleviated via SRG-Induced Autophagic Activity

**Figure 8:**
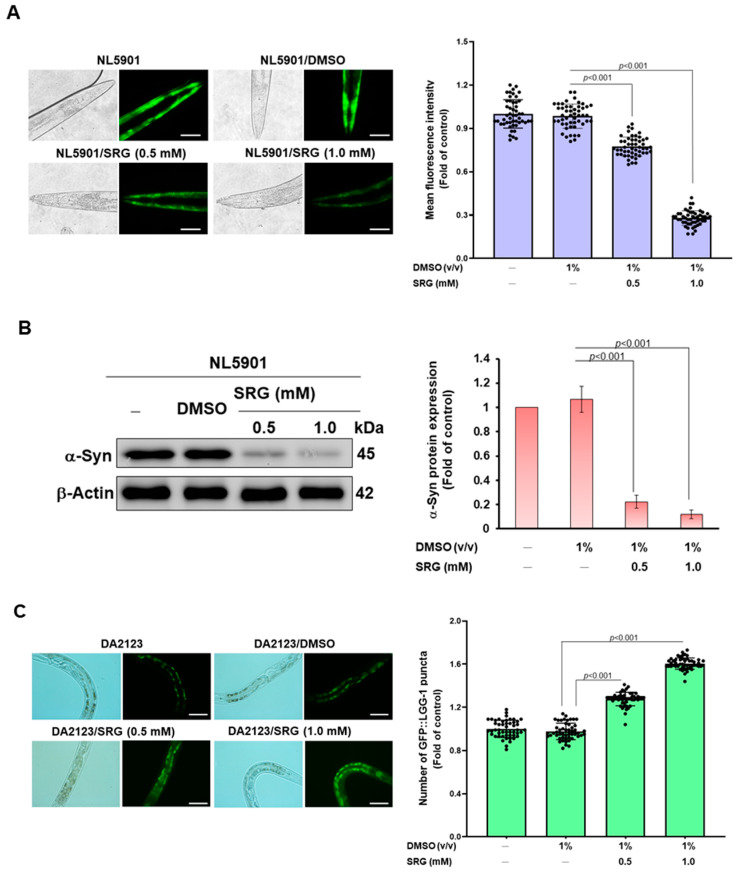
SRG-induced autophagic activity relieves α-synuclein accumulation in muscle cells of worms. (**A**) Muscle cells of NL5901 worms expressing YFP-fused human α-synuclein were used to assess α-synuclein accumulation. Fifty worms were tested in each group. The fluorescence signals of YFP were calculated via ImageJ software. (scale bar = 100 μm) (**B**) The expression of YFP-fused human α-synuclein from (**A**) was analyzed via Western blotting. The signal intensity was quantified via ImageJ software. (**C**) DA2123 worms expressing the LGG-1 promoter-inducible GFP-fused LGG-1 protein in hypodermal seam cells were used to assess autophagic activity in worms. Fluorescent punctate signals represent the formation of autophagosomes (autophagy activation) and are counted from at least 10 seam cells per worm. Fifty worms were tested in each group. (scale bar = 200 μm).

Since it was shown in cell models that SRG can promote autophagic activity, we wanted to further evaluate its effect on the accumulation of α-synuclein (the formation of Lewy bodies), the main feature of PD in vivo. We employed the NL5901 worm model that has been widely used to assess the accumulation of human α-synuclein. Its muscle cells express yellow fluorescent protein (YFP)-fused human α-synuclein. Due to the large size of the cells, it is easy to observe the accumulation of specific fluorescent proteins. The results revealed that SRG could reduce the accumulation of α-synuclein in a dose-dependent manner (Figure 8A). In the SRG (1 mM) group, compared with the DMSO group, the accumulation of α-synuclein was significantly reduced by 71.6% (*p* < 0.001 Figure 8A). We also analyzed the level of α-synuclein in worms. The results of Western blotting were consistent with the data of fluorescence quantification (Figure 8B). Compared with the DMSO group, the level of α-synuclein in the SRG (1 mM) group was significantly reduced by 88.8% *(p* < 0.001, Figure 8B).

Next, we assessed the autophagy activity in epithelial seam cells using DA2123 worms expressing a GFP-fused LGG-1 protein (homolog of human LC3) induced via the LGG-1 promoter. Its autophagic activity is reflected in aggregated puncta (green fluorescent), representing the formation of autophagosomes. The results showed that SRG treatment could enhance the autophagic activity of the worms in a dose-dependent manner (Figure 8C). Compared with the DMSO group, in the SRG (1 mM) group, the autophagic activity could be increased by 1.6-fold (*p* < 0.001 Figure 8C).

### 3.10. SRG May Protect against 6-OHDA Toxicity to DA Neurons of Worms via Strengthening the Sir-2.1 Pathway

**Figure 9:**
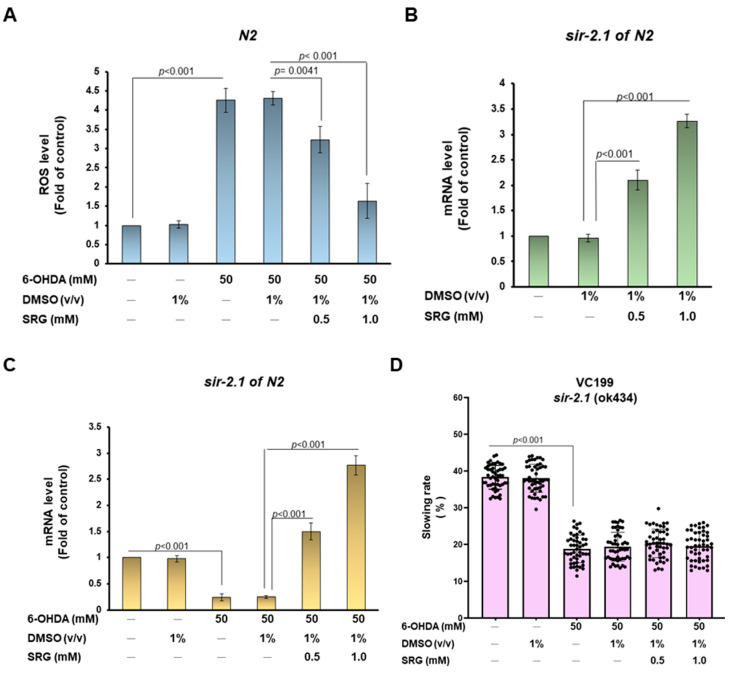
SRG pretreatment inhibited 6-OHDA-caused ROS upregulation, and promoted the *sir-2.1* pathway in N2 worms. (**A**) Thirty worms from each group were placed in the wells of a 96-well plate to detect the total ROS level using an H2DCFDA probe and a spectrophotometer. (**B**) Quantitative detection of sir-2.1 expression in worms treated with SRG via RT-qPCR. (**C**) The effect of SRG pretreatment on sir-2.1 expression in N2 worms exposed to 6-OHDA was quantified via RT-qPCR. (**D**) The VC199 strain (the mutant of sir2.1) was used to assess the effect of sir-2.1 on SRG by improving food sensitivity behaviors impaired via 6-OHDA in worms.

Because intracellular ROS levels are associated with the 6-OHDA-induced degeneration of DA neurons, we wanted to assess whether ROS production in 6-OHDA-exposed worms would be reduced via SRG pretreatment. We found a significant 4.2-fold increase in ROS levels in N2 worms in the 6-OHDA group compared with the control group (*p* < 0.001, Figure 9A). However, SRG dose-dependently reduced the level of ROS in 6-OHDA-exposed worms. In the SRG (1 mM) group, compared with the 6-OHDA (DMSO) group, ROS levels were reduced by 64.3% (*p* < 0.001, Figure 9A). Finally, we wanted to confirm whether the neuroprotective ability of SRG in 6-OHDA-exposed worms was related to SIRT1. The worm orthology similar to human *SIRT1* is *sir-2.1*. The results of the RT-qPCR analysis showed that SRG could enhance the expression of *sir-2.1* in a dose-dependent manner. The expression of *sir-2.1* was augmented by 3.4-fold under SRG (1 mM) treatment compared with the DMSO group (*p* < 0.001, Figure 9B). The expression of *sir-2.1*, which was significantly suppressed under 6-OHDA treatment (*p* < 0.001, Figure 9C), was significantly enhanced by 11.1-fold (*p* < 0.001, Figure 9C) under the treatment of SRG (1 mM). Furthermore, we used the *sir-2.1* mutant worm strain VC199 for food sensitivity behavioral tests. Results showed that slowing rates reduced via 6-OHDA exposure were not restored via SRG pretreatment. These results confirmed that SRG improved the toxicity of 6-OHDA on DA neurons and the accumulation of α-synuclein in vivo partly through the upregulation of the *sir-2.1* pathway.

## 4. Discussion

This study demonstrated that SRG could alleviate 6-OHDA-caused oxidative stress and apoptosis in SH-SY5Y cells. Similarly, in the *C. elegans* animal model, the 6-OHDA-induced degeneration of DA neurons, dopamine-mediated food-sensitive behavioral abnormalities, and shortened lifespan were improved. Further investigation revealed that the therapeutic potential of SRG is partly through the induction of autophagy. Neuronal toxins such as 6-OHDA can disrupt the important cellular program, leading to abnormal protein aggregation/denaturation and mitochondrial dysfunction in neurons. Exposure to high concentrations (100 μM) of 6-OHDA decreased autophagy in SH-SY5Y cells, while pretreatment with SRG not only reversed this effect but also enhanced autophagy activity.

Autophagy eliminates aged abnormal proteins or damaged cytoplasmic organelles [42]. The 6-OHDA causes the downregulation of autophagy-related proteins, including PI3 kinase (PI3K) p100, Beclin-1, and Atg7. SRG pretreatment not only reversed this result but also enhanced the expression of these autophagy-related genes. The macroautophagy pathway can be controlled via different PI3K signals. Among them, the upregulation of the class I PI3K/Akt pathway can inhibit macroautophagy. mTOR, a downstream target of this pathway, regulates Atg proteins, thereby preventing autophagosome formation. Here, rapamycin can inhibit the activity of mTOR, thereby promoting autophagy. Therefore, we investigated whether mTOR activity is related to SRG-mediated autophagy. We found that SRG did not increase the level of phosphorylated mTOR protein. Conversely, a rise in the class III PI3K complex formed via the PI3 kinase p100 subunit, p150 subunit, and Beclin-1 induces macroautophagy [45]. The Beclin-1/class III PI3K complex plays an important role in autophagy initiation and autophagosome formation/maturation. Here, we found that the pretreatment of cells with SRG augmented the protein expression of PI3 kinases p100, Beclin-1, and Atg7. Likewise, it increased the LC3-II/LC3-I ratio and autophagophore establishment. Therefore, Beclin1/class III PI3K signaling is partly responsible for the upregulation of autophagy via SRG. Additionally, clearance of α-synuclein aggregated in the neuronal cell line can be enhanced via overexpression of Beclin-1 [46]. It was also observed in our *C. elegans* model that SRG promotes autophagy and reduces α-synuclein accumulation. These results indicated that SRG could inhibit 6-OHDA-induced apoptosis and α-synuclein accumulation in PD models via enhancing the expression of Beclin-1 and promoting autophagy.

Beclin-1 is an evolutionarily conserved protein that functions as a positive regulator in autophagosome biogenesis. In addition to regulating expression, some modifications can affect the activity of Beclin-1, including ubiquitination, SUMOylation, and acetylation. For example, phase kinase-associated protein 2 (SKP2) as an E3 ligase can promote ubiquitin-mediated proteasomal degradation of Beclin-1 [47]. Beclin-1 can be SUMO3-conjugated mainly at lysine residues 380 via a protein inhibitor of activated STAT 3 (PIAS3) and de-SUMOylated via SUMO-specific peptidase 3 (SENP3). SUMOylation increases upon cell starvation, promoting the interaction of Beclin-1 with other components to enhance autophagosome formation, thereby increasing class III PI3K activity. Conversely, SENP3 deSUMOylates Beclin-1, impairing the formation or stability of the Beclin-1-class III PI3K complex, thereby inhibiting the class III PI3K activity [48]. Beclin-1 is acetylated via the E1A binding protein p300 (Ep300) and deacetylated via SIRT1 (Sirtuin 1) at lysine residues 430 and 437. The acetylation of Beclin-1 inhibits autophagosome maturation, resulting in impaired autophagic flux [16,49]. In this study, we found that SRG did not alter the ubiquitination and SUMOylation of Beclin-1. However, immunoprecipitation analysis showed that SRG reduces the acetylation of Beclin-1 caused by 6-OHDA. We further analyzed the activity of the acetylating enzyme p300 and the deacetylating enzyme SIRT1 of Beclin-1. The results indicated that SRG did not affect the expression of p300 but could induce the expression of SIRT1 to promote the deacetylation of Beclin-1 and then augment the activity of autophagy. In the future, we will conduct a whole genome microarray analysis [50,51] when evaluating the effectiveness of SRG on dopaminergic neurons derived from induced pluripotent stem cell lines of PD patients. The results of the microarray analysis will provide a detailed determination of changes in gene expression profiles and lead us to a deeper understanding of the precise mechanism of SRG in preventing the cytopathology of PD.

Current studies have shown that in addition to affecting Beclin-1 activity, SIRT1 can also promote autophagy via modulating other autophagy-related proteins such as AMPK, LC3, mTOR, and FoxO [15]. In terms of maintaining and regulating mitochondrial function and inhibiting oxidative stress, SIRT1 can promote PGC-1α to be in a deacetylated state and maintain a constant level of PGC-1α to achieve this [52]. Furthermore, SIRT1 can also regulate PD-associated neuroinflammation [53]. For example, the reduction of transcriptional activity via the deacetylation of the nuclear factor-κB leads to the downregulation of nitric oxide synthase, tumor necrosis factor-α, and interleukin-6 levels [54]. SIRT1-dependent deacetylation of heat shock factor 70 promotes the expression of the heat shock protein 1, leading to the degradation of α-synuclein inclusion bodies [55]. Previous studies have shown that SIRT1 activity is reduced in patients with PD. This can lead to a reduced ability of the patient to resist neuronal damage [56]. SIRT1-specific SNPs have also been shown to be associated with PD risk [57]. According to the extensive neuroprotective effect of SIRT1, some SIRT1 promoters, including resveratrol, have been proven in vitro and in vivo to prevent and treat DA neuron damage. Therefore, SIRT1 can be used as a potential target in the development of PD therapy [15].

Further studies found that SRG-induced SIRT1 mRNA expression did not coincide with a significant increase at the protein level. Therefore, we believe that SRG may affect the post-transcriptional modification of the SIRT1 gene, such as regulating the expression of miRNAs targeting SIRT1. According to the results of RT-qPCR analysis, we found that the expression of miR-34a was greatly reduced via SRG treatment. The transfection of SH-SY5Y cells with the miR-34a inhibitor can significantly improve the expression of SIRT1 under 6-OHDA exposure. In this case, SRG pretreatment did not significantly increase SIRT1 expression. Furthermore, the transfection of cells with miR-34a mimics further inhibited SIRT1 protein expression upon exposure to 6-OHDA and abolished the character of SRG to reverse the inhibition of 6-OHDA on SIRT1 expression.

Aging and metabolic syndrome are associated with the increased expression of miR-34a [58]. miR34-a represses the nicotinamide phosphoribosyltransferase gene (Nampt) expression, leading to reduced levels of nicotinamide adenine dinucleotide (NAD) and affecting SIRT1 activity [59]. The pro-inflammatory transcription factor NF-κB can bind to the gene promoter region to increase the expression of miR-34a [60]. Similarly, the upregulation of miR-34a can also enhance the activity of NF-κB [61]. In addition, the overexpression of miR-34a can promote the apoptosis of cancer cells [25], hinder the cell cycle, and inhibit metastasis [62] and angiogenesis [58]. Therefore, the inhibition of miR-34a via SRG can not only increase the expression of SIRT1 to induce the activity of autophagy but also strengthen the ability of cells to resist apoptosis, anti-aging, and promote regeneration. Furthermore, PD is associated with chronic inflammation [63] and diabetes [64]. The downregulation of miR-34a via SRG can further improve the effects of chronic inflammation and diabetes on the progression of PD.

Some synthetic or natural bioactive molecules can induce autophagy and may be used to improve or treat PD. For example, semaglutide induces autophagy in SH-SY5Y cells and relieves oxidative stress and mitochondrial dysfunction caused by 6-OHDA [65]. Ferulic acid can activate autophagy to degrade the human α-synuclein expressed in *C. elegans* and prevent H_2_O_2_-induced apoptosis in PC-12 cells [66]. Piperin promotes autophagic flux via enhancing the autophagosome–lysosome membrane fusion and causes degradation of pathological α-synuclein, alleviating the olfactory deficit and delaying the motor deficit in transgenic mice [67]. The celastrol of *Tripterygium wilfordii* reverses rotenone-stimulated α-synuclein aggregation and apoptosis by elevating the ratio of LC3-II to LC3-I [68]. SRG is a new member of natural products with properties that can enhance the activity of autophagy.

## 5. Conclusions

Our results suggest that SRG ameliorates oxidative stress and apoptosis of DA neurons induced via 6-OHDA neurotoxins in vitro and in vivo via inhibiting miR-34a to strengthen the SIRT1/beclin1/autophagy axis. Furthermore, SRG alleviated the accumulation of α-synuclein. Therefore, SRG deserves further evaluation for its value as a drug candidate for preventing neurodegeneration such as PD.

## Data Availability

All data used and analyzed during this study are available from the corresponding author upon reasonable request.
